# Opti-nQL: An Optimized, Versatile and Sensitive Nano-LC Method for MS-Based Lipidomics Analysis

**DOI:** 10.3390/metabo11110720

**Published:** 2021-10-21

**Authors:** Angela Cattaneo, Giuseppe Martano, Umberto Restuccia, Laura Tronci, Michele Bianchi, Angela Bachi, Vittoria Matafora

**Affiliations:** 1 Cogentech SRL Benefit Corporation, 20139 Milan, Italy; angela.cattaneo@cogentech.it (A.C.); michele.bianchi@cogentech.it (M.B.); 2 Institute of Neurosciences (CNR), 20133 Milan, Italy; giuseppe.martano@in.cnr.it; 3 The FIRC Institute of Molecular Oncology (IFOM), 20139 Milan, Italy; umberto.restuccia@adienne.com (U.R.); laura.tronci@external.ifom.eu (L.T.); vittoria.matafora@ifom.eu (V.M.); 4 ADIENNE Pharma & Biotech, 20867 Caponago, Italy; 5 Division of Genetics and Cell Biology, IRCCS-San Raffaele Scientific Institute, Molecular Basis of Cystic Kidney Diseases, 20132 Milan, Italy

**Keywords:** lipidomics, nano-LC-MS/MS, lipid species, quantitative analysis, sensitivity

## Abstract

Lipidomics is the comprehensive analysis of lipids in a given biological system. This investigation is often limited by the low amount and high complexity of biological samples, therefore highly sensitive lipidomics methods are required. Nanoflow-LC/MS offers extremely high sensitivity; however, it is challenging as a more demanding maintenance is often needed compared to conventional microflow-LC approaches. Here, we developed a sensitive and reproducible lipidomics LC method, termed Opti-nQL, which can be applied to any biological system. Opti-nQL has been validated with cellular lipid extracts of human and mouse origin and with different lipid extraction methods. Among the resulting 4000 detected features, 700 and even more unique lipid molecular species have been identified covering 16 lipid sub-classes, while 400 lipids were uniquely structure defined by MS/MS. These results were obtained by analyzing an amount of lipids extract equivalent to 40 ng of proteins, being highly suitable for low abundant samples. MS analysis showed that theOpti-nQL method increases the number of identified lipids, which is evidenced by injecting 20 times less material than in microflow based chromatography, being more reproducible and accurate thus enhancing robustness of lipidomics analysis.

## 1. Introduction

Lipids are one of the major constituents of biological systems. They are essential for biological membrane formation and energy storage and participate to many cellular signaling processes [1]. Maintaining lipid metabolic homeostasis is crucial for every organism. It has been reported that lipid dysregulation is linked to many diseases such as obesity, chronic inflammation, cardiovascular and neurodegenerative disorders, diabetes, Parkinson and cancer [2]. Identification and quantification of lipids have therefore become an important need in both biomedical and basic research. The systematic analysis of the overall lipid composition in a given system is called lipidomics, which is one of the branches of metabolomics. The most suitable method for lipidomics analysis is mass spectrometry. Technological advances in this field, such as high mass resolution combined with high mass accuracy, faster scan rates and high reproducibility, have facilitated the application of MS-based techniques both in quantitative and qualitative analysis of biomolecules [3]. MS-based lipidomics analysis is able to generate comprehensive, quantitative and reproducible data with high efficiency and robustness. However, this approach displays several analytical obstacles, mainly related to sample complexity.

According to their chemical structure, lipids are classified into eight categories: fatty acyls, glycerolipids, glycerophospholipids, sphingolipids, sterols, prenol lipids, saccharolipids, and polyketides [4]. All categories are further subdivided into lipid classes and lipid subclasses. The number of naturally occurring lipids is calculated around 100,000 or even more species, being far from the compounds annotated so far in lipids databases [5]. Lipid complexity leads to many potential mass spectral overlaps of lipid molecular ions and molecular adduct ions, therefore isobaric species are commonly encountered in MS analysis. Shotgun lipidomics, coupled with high mass resolution instruments, is the principal MS based approach used for lipids analysis. Either Direct Infusion (DI) of lipid extract or LC-based separation coupled with MS detection, are generally used for this analysis [6]. The term ‘shotgun lipidomics’ generally refers to DI-MS. This method involves intra-source separation of lipid classes without any prior chromatographic separation, and the subsequent application of precursor ion and neutral loss scans of polar head group and fatty acid moieties for lipid identification [6,7]. Compared to LC-based method, DI-MS is stable in lipid quantitation, simpler in sample handling and faster in sample analysis. However, the disadvantage is that DI-MS spectra are bias towards the most abundant or easily ionized lipids. This limitation can be overcome by targeting one class of analytes at a time using dedicated protocols or by performing specific derivatizations of a defined functional group [8]. Furthermore, DI is hampered by the presence of isobaric species which might be circumvented by LC-MS based approaches which offer an increased dynamic range and an additional level of identification based on chromatographic retention time. The two most widely used chromatographic approaches in LC-MS are reversed-phase chromatography and hydrophilic interaction liquid chromatography (HILIC) [9]. Reversed-phase LC separates lipids based on hydrophobic interaction between the fatty acyl groups and the stationary phase (the retention time increases as the number of carbons increases and decreases as the number of double bonds increases). HILIC, instead, separates lipids mainly on the basis of the characteristics of their polar head groups. LC-based separation coupled to MS detection provides molecular specificity using exact mass measurement, structural information obtained by MS/MS and retention time to assign a detailed chemical structure to each lipid identification. Depending on the type of MS/MS analysis there are different levels of information that can be obtained. The first level of annotation for lipid identification is the lipid membership class and its rough composition, which is based on the sum of carbon atoms and double bonds present. For instance, PC 36:2 is phosphatidylcholine with 36 carbon atoms and 2 double bonds. Additional information on the length of saturated and unsaturated fatty acids increases the annotation level to lipid molecular species: e.g., PC 14:1/22:1 which is phosphatidylcholine with a 14 carbons alkyl chain in sn1-position carrying 1 insaturation and with a 22 carbons alkyl chain in sn2-position carrying 1 insaturation. Finally, molecular lipids are fully defined when elucidation of structural features such as the position of the double bonds are also detected: e.g., PC 14:1(9Z)/22:1(13Z) (ref. HMDB0007920) which is phosphatidylcoline with a 14 carbons alkyl chain in sn1-position carrying 1 insaturation at position 9 and with a 22 carbons alkyl chain in sn2-position carrying 1 insaturation at position 13 [10]. The level of annotation is strictly dependent on the availability of appropriate software tools, on the completeness of lipids MS/MS databases, on the quality of in silico databases such as LIPID MAPS and, importantly, on the data acquisition method and on the quality of data. It has to be mentioned that there are also recent alternative techniques, such as ion mobility-mass spectrometry (IM-MS), able to refine the structural information on the fatty acyl composition identifying unsaturated double bond positions and the cis-trans geometry of the lipid chain [11]. The majority of LC-based lipidomics studies are currently performed using micro, narrow- or analytical bore LC columns. Traditionally, microLC systems are preferred because they are considered more robust at the expense of sensitivity, whereas nanoLC systems are not routinely used because they are more prone to leaks and dead volumes which cause peak broadening, and to fast clogging of columns and emitters due to the formation of salt crystals [12]. Nevertheless, nano-scale analytics displays higher efficiency and sensitivity, offers low organic solvent consumption and reduced waste and is essential when the biological material is limited, for example with small amount of tissue biopsies or rare cell subpopulations [13,14]. Moreover, it can be easily applied for multiple omics applications that uses small fractions of diluted samples [15]. Here, starting from the investigation of mobile phases propensity to form salt crystals, we have developed a robust and fast nano LC-MS/MS method, named Opti-nQL (Optimized-nano Quantitative Lipidomics) method, able to achieve a comprehensive and high-quality analysis of lipid species. We further showed that Opti-nQL is compatible with alternative lipid extraction methods and with different biological samples.

## 2. Results and Discussion

### 2.1. Method Development

#### Lipid Extraction and LC Optimization

**Figure 1:**
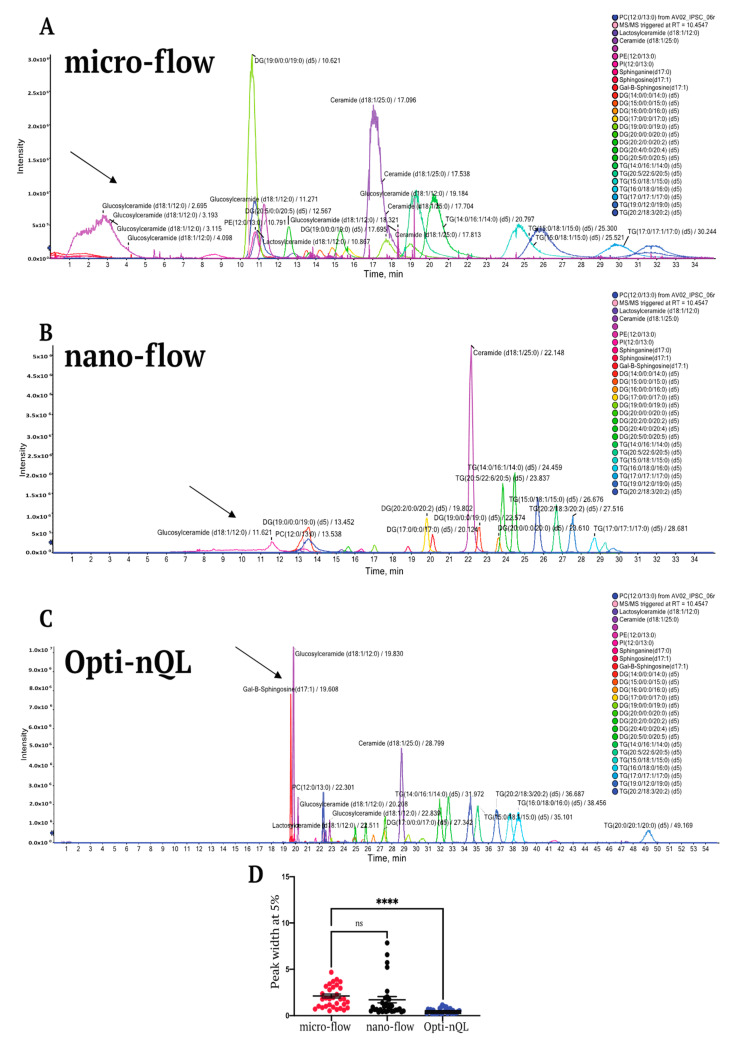
LC chromatographic profiles of the standards mixture. (**A**) Extracted ion chromatogram (EIC) of internal standards (IS) analyzed with micro-flow workflow. (**B**) EIC of IS analyzed in nano-flow workflow. (**C**) EIC of IS analyzed in optimized nano workflow (Opti-nQL). (**D**) Peak width at 5% of the peak height for all IS analyzed in microflow, nanoflow and Opti-nQL. *t*-test **** *p*-value < 0.0001. Notably, IS concentration injected in microflow is 1/12 of the amount (pmol) listed in Table 1 while for nanoflow it is 1/250.

Lipid extraction was performed by using the Folch-chloroform based extraction [16], which is one of the most commonly used methods for extracting lipids from biofluids and tissues and recently used also for low sample amount [17]. In our protocol, an amount of sample equivalent to 10 µg of proteins was used for lipid extraction. Following the guidelines of the Lipidomic Standards Initiative (LSI) [18], Internal standards were spiked into the samples as recommended by Wang et al. [19]. The extracted internal standards mixture was used for LC optimization. Compared to micro LC, nano LC suffers the occurrence of leaks and clogging due to the formation of salt crystals, reducing chromatographic column lifetime and analytical reproducibility. Therefore, we first analyzed the suitability of mobile phases commonly used for lipids LC separation [20,21] with the aim to select the one with the lower propensity to form salt crystals, hence best suited for nanoflow analysis. The chosen chromatographic stationary phase was a C-18 reversed-phase, which separates lipids based on their nonpolar fatty acyl moieties. According to the equivalent carbon number theory [22], lipids of the same class are separated depending on the number of fatty acyl carbons and double bonds. We tested two mobile phase compositions, such as: Phase A1 = CH_3_CN:CH_3_OH:H_2_O 45:45:10; 5 mM NH_4_COOH; 0.1% HCOOH and Phase B1 = IPA: H_2_O 90:10; 5 mM NH_4_COOH; 0.1% HCOOH and as alternative Phase A2 = CH_3_CN:CH_3_OH:H_2_O 45:45:10; 5 mM NH_4_COOCH_3_; 0.1% HCOOH and Phase B2 = IPA: H_2_O 90:10; 5 mM NH_4_COOCH_3_; 0.1% HCOOH. We dried 10 mL of the above described phases on a glass tube and we analyzed salt crystals deposits. From our test, the presence of NH_4_COOH determines an increased accumulation of salt crystals when compared to phases containing NH_4_COOCH_3_ (Figure S1). The mobile phases were tested also in the LC system and as expected, we observed that the presence of NH_4_COOH generates frequent column clogging which was not observed in the presence of NH_4_COOCH_3_ (data not shown). Therefore, based on these observations we decided to use the mobile phases A2 and B2 in the Opti-nQL method. We started to set up the method by using microflow LC and, once the optimal chromatographic conditions were found, we moved to nanoflow LC, with further optimization. In order to do so, Ekspert nanoLC400 (Eksigent) system, equipped with nano or micro flow module for nano and micro chromatographic analysis, was used. For the chromatographic separation of lipids in microflow, we used reversed phase chromatography with an analytical column HALO C18 90A 0.5 × 50 mm, 2.7 μm particle size, coupled with the solvent system (organic/aqueous) that we have previously selected, and operated at 10 μL/min. Conversely, for the nanoflow separation, we used reverse phase chromatography with an analytical column Kinetex EVO C18 100A, 75 μm × 10 cm, 1.7 μm particle size, with the same mobile phase used for microflow at a flow rate of 150 nL/min. By using a 35 min gradient, we compared, in micro and nano workflow, the extracted internal standards mixture. The concentration of the sample injected in the nano configuration was 20 times lower than the concentration injected in microflow. We were able to separate all the internal standards (IS) both in micro and nano configuration with the exception of glucosylceramide that shows a broader peak and is almost invisible in the chromatogram (Figure 1A,B).

We then optimized the chromatographic gradient to obtain sharper peaks also for glucosylceramide, GlcCer (d18:1/12:0) (Figure 1C). In particular, we removed methanol from both buffer A and from the resuspension buffer, and we increased the length of the chromatographic separation of 20 min (Figure 1C). Notably, the chromatographic resolving power was improved by nano flow as all standards showed sharper elution peaks. Moreover, the sensitivity of the method resulted highly increased, as many more lipids were stochastically selected for MS/MS analysis with reduced spectral interferences (Figure 1D). After the optimization of the nanoflow method, we assessed the linearity of the peak areas for all the standards. In particular we tested four sequential dilutions from 1 to 10-fold, where 1 corresponds to an amount of IS equivalent to 1/250 of the IS concentration listed in Table 1. For all the standard we also reported LOD and LOQ (Table S1), whose values are globally lower compared to recent lipidomics published works [23,24]. Our data demonstrate that the standards intensities increase proportionally with the injected amount, as evident by observing the total ion current (TIC) of the analysis (Figure S2). All the standards showed high linearity, reaching an average R^2^ of 98% (Figures S2 and S3). Moreover, both intra- and inter-day variability are very low indicating that the Opti-nQL method is highly stable (Figure S4). In particular, IS were analyzed twice the same day or in different days spanning from 0 to 75 days after the first run. Both peak areas and RT values are highly reproducible during time attesting the robustness of the presented method (Figure S4).

### 2.2. Lipidomics Analysis of Mammalian Cells

#### 2.2.1. Semi-Targeted Lipidomics Analysis

**Figure 2:**
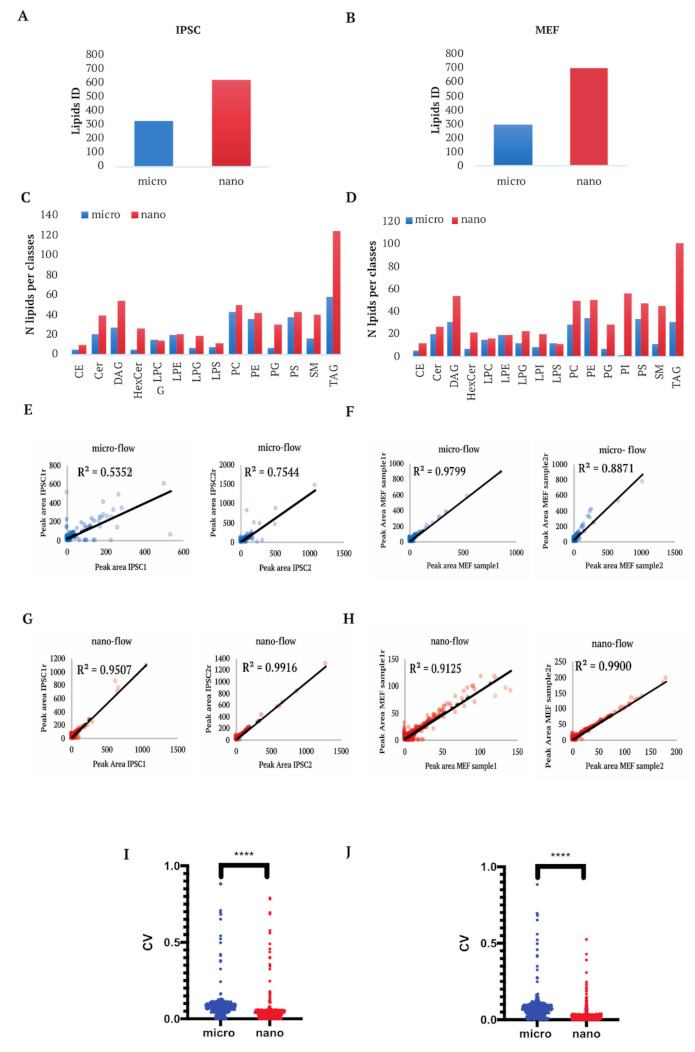
Lipid identification in micro and nano workflow. (**A**) Total lipids identified in micro and nano-flow methods from 2 biological and 2 technical replicates of each IPSC sample. (**B**) Total lipids identified in micro and nano-flow methods from 2 biological and 2 technical replicates of each MEF sample. (**C**) Total lipids per class identified by micro and nano-flow in IPSC. (**D**) Total lipids per class identified in micro and nano-flow. (**E**) Technical reproducibility of the peaks area of the lipids identified in IPSC with the micro-flow method. (**F**) Technical reproducibility of the peaks area of the lipids identified in MEF by the micro-flow method. (**G**) Technical reproducibility of the peaks area of the lipids identified in IPSC samples with the nano-flow method. (**H**) Technical reproducibility of the peaks area of the lipids identified in MEF samples with the nano-flow method. (**I**) Coefficient of variation (CV) of the peak areas of all the lipids identified in all the IPSC replicates in micro and nano flow methods. (**J**) Coefficient of variation (CV) of the peak areas of all the lipids identified in all MEF replicates in micro and nano flow methods. *t*-test **** *p*-value < 0.0001.

To assess the performance of Opti-nQL with complex biological samples, we used lipids extracts from human (IPSC, Induced Pluripotent Stem Cell) and mouse (MEF, Mouse Primary Embryonic Fibroblasts) cell lines. The same samples were analyzed both by micro and nanoLC in order to evaluate the performance of both workflows. LC-MS/MS analyses were performed exclusively in positive mode as we did not observe a reasonable improvement in lipid coverage by combining positive and negative acquisitions (data not shown). Briefly, two biological replicates with two technical replicates, were acquired for each cell line to assess the reproducibility of the method. Spectra were analyzed by LipidView (AB-SCIEX) in a semi-targeted approach, by selecting lipid classes, decided by the user, to be searched in the raw data. The selected lipid classes in our study were: Glycerophospholipids, Sphingolipids, Glycerolipids and Sterol Lipids. To asses the quality of the extraction and the matrix effect we evaluated qualitatively and quantitatively the IS mixture spiked in the samples. The nanoflow and microflow analyses of the areas of the spiked standards in extracts from Mouse Embryonic Fibroblasts (MEFs) and human IPSC cells are reported in Figure S5. As expected, all standards were detected and quantified in both LC configurations. Indeed, these results reflect the high efficiency of the Folch’s lipid extraction method as it is reported that the recovery for most prominent lipid classes (such as PC, SM, PE, TAG, and DAG) is above 90% while lipid classes with more polar and partially negative charged headgroups (such as PI, PG, PA, and lyso-lipids) have recoveries of 60–70% [25]. Moreover, the comparison of IS peak area analyzed alone or spiked in MEFs and IPSC extracts shows that most of the lipid classes do not exhibit matrix effects (Figure S6) as they were equally detected. As mentioned before, LipidView identifies lipid species on the basis of class specific precursors ions, class-indicative fragment ions, fatty acyl product ions and neutral loss ions. Overall, the micro-flow analysis led to the identification of lipid species across 16 lipid classes with 295 and 330 total lipid species identified in MEFs and human IPSC, respectively (Figure 2A,B), with an average of 182 and 251 lipids identified and quantified per run (Figure S7A).

Conversely, in nanoflow, injecting 20 times less material, we almost doubled the number of total identified lipid species in both MEF (698) and human IPSC (608) (Figure 2A,B), where an average of 559 and 451 lipids was identified and quantified per run (Tables S2–S5 and Figure S7A). Differences in lipid IDs among each run, in both micro- and nano-flow, are due to the presence of missing values, which are a common problem in omics approaches. Indeed, missing values occur in datasets for several reasons: lipid amount below the limit of detection; stochastic nature of precursor selection in data dependent acquisition methods; missing peak picking by the software and biological variability across different samples [26]. In line with the total identified lipids, also core lipids across replicates are doubled in nano-flow compared the micro-flow datasets (Figure S7B). Therefore, the optimized nanoLC method shows better performances in terms of increased number of total lipids identified and quantified well distributed among each lipid class (Figure 2C,D and Tables S2–S5), but also in terms of improved technical reproducibility (Figure 2E–H) and decreased coefficient of variation (Figure 2I,J). The improvements listed above confirm the nanoLC method as the best choice for a comprehensive lipidomics analysis as it enables robust quantitation together with high reproducibility and sensitivity. Furthermore, we analysed the lipidomics profile of melanoma WM115 cells and we obtained a comparable number of lipids identified and quantified (Table S6), further attesting the reproducibility of the nanoLC method across different types of samples. Indeed, we extensively utilized the above described nanoLC analytical method as demonstrated by recent publications [27,28,29].

**Figure 3:**
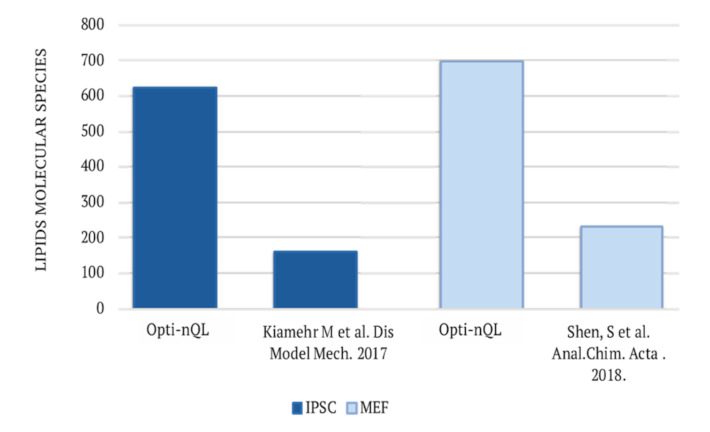
Comparison of Opti-nQL method with already published lipidomics analysis performed on the same cell lines. Opti-nQL leads to a higher number of identified lipids.

Of note, Opti-nQL led to a higher number of identified lipid species if compared with published lipidomics studies performed on MEFs [30] and IPSC [31] cells by using micro-flow LC (Figure 3).

#### 2.2.2. Untargeted Lipidomics Analysis

The analysis with LipidView^TM^, although user-friendly, shows some limitations as it is semi targeted and it is based on matching precursor and fragment ion masses to the in silico tandem MS database embedded in the software. In details, LipidView^TM^ enables the identification of lipid species and lipid classes by matching specific lipid head groups precursor ions, fatty acid characteristic fragments and neutral losses. The unambiguous assignment of lipids to a precise structure is essential to understand their biological implications [32]. Within the same class, lipid species differing for the position of the double-bond moiety or for the relative position of the acyl group, may belong to different biological pathways, and therefore their accurate identification is pivotal to elucidate potential physiological links.

**Figure 4:**
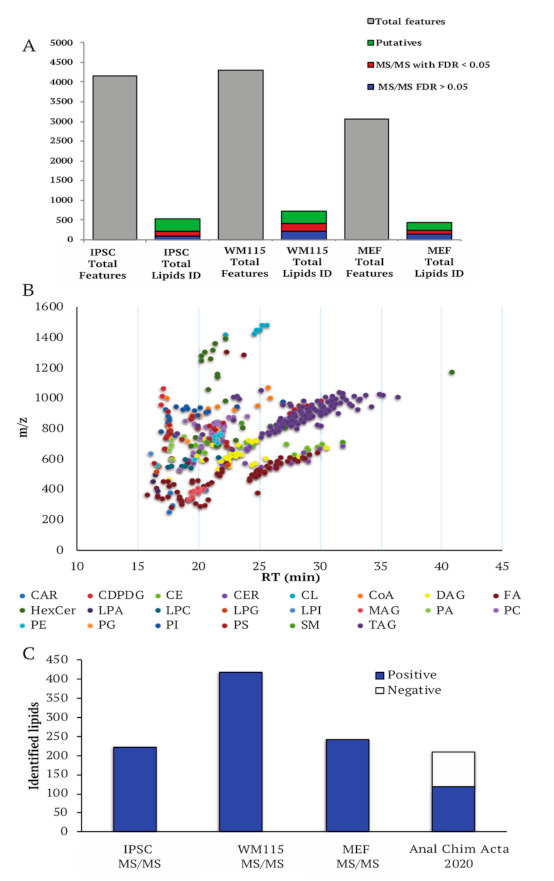
Untargeted lipidomics analysis by SMFinder. (**A**) Number of features and total lipids identified by exact mass and MS/MS match with or without FDR filter in all three datasets: MEF, IPSC, and WM115 cell lines. (**B**) Lipid classes distribution of all lipids identified by SMFinder along the gradient in dependence of *m/z*. (**C**) Comparison of lipids identified by MS/MS match in this study and in a recently published work using nanoflow-based lipidomics method.

In order to have a more comprehensive and specific analysis of the lipidome profile, we further analyzed the samples by SMFinder [27], an in-house developed software able to match MS and MS/MS spectra with available lipidomics databases. Differently from the library present in LipidView^TM^, lipidomics databases used in SMFinder are library of empirical spectra or in silico derived lipids fragmentations. In all the analyzed cell lines, 3000–4000 MS features were detected above the intensity threshold being shared in at least three out of four replicates of each dataset (two technical, two biological replicates) (Figure 4).

Overall, 30% of them were fragmented. Given that the detected MS features do not correspond to the actual number of lipids, as multiple adducts corresponding to the same lipid might be present, we removed all redundant and contaminant MS signals. We analyzed the above features by identifying lipids with different levels of confidence, from the less stringent level, based exclusively on matching to the exact mass of known lipids listed in LipidMaps database, to the most confident criteria based on matching both exact mass and MS/MS fragments to the ones contained in currently available lipids databases, with or without applying FDR filtering [27]. From the analysis of all the datasets, a total of 500–700 lipids matched to biological relevant lipids exclusively by exact mass (putative lipids) (Figure 4A). A total of 250–450 features matched to known lipids in the lipidomic databases at both by exact mass and MS/MS fragmentation, half of them also passed the FDR filter (Figure 4A, Tables S7–S9). The identified lipids span over 20 lipid classes which were well distributed along the LC gradient (Figure 4B). In particular, each lipid class is eluted reflecting the structural differences due to its specific chemical composition. For instance, hydrophilic lipids, such as diacylglycerophospholipids and phospholipids species (PC, PE, PG, PI, PS) are eluted first in reversed-phase chromatography, while hydrophobic TAG are at the end of the chromatographic separation (Figure 4B).

Compared to a recent nanoflow-based method [17] that used the same lipid databases of this study for lipid identification, Opti-nQL leads to a higher number of identified lipids with the most confident criteria (MS/MS level) (Figure 4C). Generally, the rate of identification by MS/MS strongly depends on the availability of a lipids database. Differently from other omics approaches, lipidomics analysis suffers the limited number of MS/MS data contained in the databases thus limiting the unique identification of lipids. Moreover, the available lipids databases are highly redundant, as the same lipid displays multiple entries depending on the type of parameters used for the analysis, such as MS instruments, ion polarity, collision energy and the formation of different adducts. The presence of these redundancies might influence lipid identification based on MS/MS calculated FDR, as the same lipid with different adducts will produce a similar fragmentation pattern.

In conclusion, as more than 3000 features were distinctly detected, we believe that, once libraries will be updated, Opti-nQL may lead to many more identifications.

### 2.3. Opti-nQL Is Compatible with Alternative Lipid Extraction Methods

**Figure 5:**
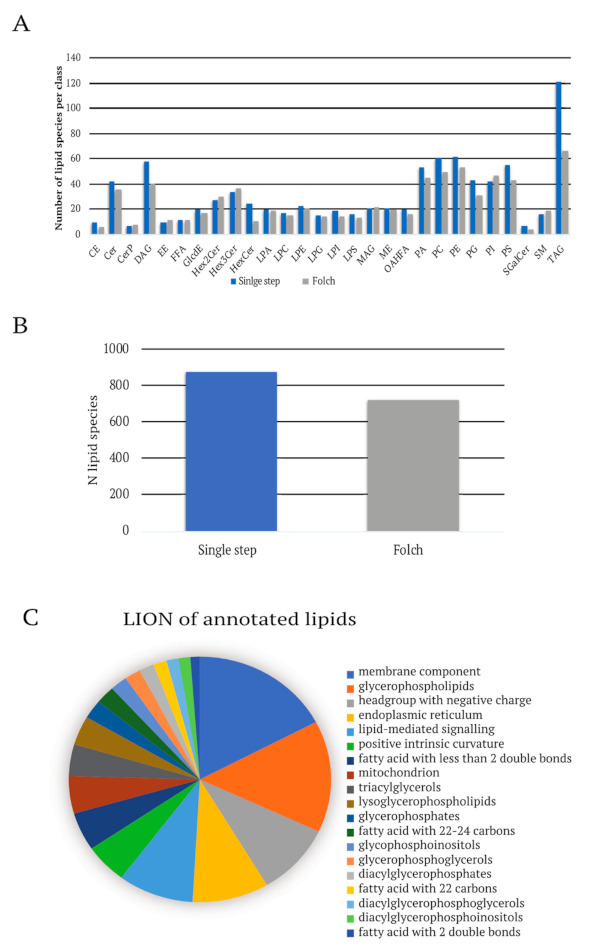
Lipidomic analysis with new lipids extraction method in IGR37cell line. (**A**) Lipids species identified per class in IGR37 cells with single-step and Folch extraction method. (**B**) Total number of lipid species identified in IGR37 cells with single-step and Folch extraction method. (**C**) Lipid ontology enrichment analysis performed with LION [35] of the lipid species identified in IGR37 cells.

Even though the Folch method is efficient and widespreadly used, other extraction methods have been reported to lead to higher yield [33,34]. Thus, we tested the performance of Opti-nQL using a different extraction method, to further demonstrate the versatily of Opti-nQL. In particular, we used a single-step lipid extraction modifying Folch method and applied it to the lipidomics analysis of IGR37 human melanoma cell line. We obtained an increase in identified and quantified lipids of 20%, from 700 to 900 lipid ID, spanning on 25 lipid classes and covering the most represented ontologies of the cell (LION analysis) (Figure 5). The faster and modified lipid extraction coupled with the Opti-nQL analysis increases sensitivity and comprehensiveness of lipid coverage. Furthermore these results additionally point to the wide applicability of the proposed method.

## 3. Materials and Methods

### 3.1. Materials

Lipid standards and SPLASH^®^ LIPIDOMIX^®^ Mass Spec Standard were purchased from Avanti Polar Lipids (Alabaster, AL, USA); solvents LCMS grade from Carlo Erba Reagents (Milan, Italy); reagents and components of buffer solutions were from Sigma-Aldrich (Darmstadt, Germany) in analytical grade or higher purity and the BCA protein assay kit was purchased from Thermo Scientific (Rockford, IL, USA).

### 3.2. Biological Sample Collection and Cell Lysis

The cell lines analyzed were: Induced Pluripotent Stem Cells (IPSC, from Telethon Genetic Biobank Network, Rome/Milano, Italy); Mouse Embryonic Fibroblasts (MEF, approval code N° 614/2015-PR, from Italian Ministry of Health); melanoma cells: WM115 (from IZSBS, ID BS-TCL74) and IGR37 (from DSMZ, ID ACC 237). Cells were cultured and collected as described previously [27,28,29]. The equivalent of 1–3 × 10^3^ cells were centrifuged at 800× *g* for 3 min and pellets were washed with 5 mL of PBS, frozen and stored at −80 °C. Cells pellets were resuspended in 250 µL of ammonium bicarbonate 150 mM and mechanically disrupt-ed by passing 20 times through a 26 G syringe needle. Samples for lipidomics analysis were analyzed either fresh or snap frozen in liquid nitrogen and stored at −80 °C until further processing.

### 3.3. Proteins Extraction and Quantification

Proteins were extracted form 20 μL of ammonium bicarbonate resuspended pellets by adding 5 μL of lysis buffer (10% NP40, 2% SDS in PBS) and quantified by BCA protein assay kit.

### 3.4. Lipids Extraction

Lipids were extracted starting from an equivalent of ammonium bicarbonate resuspended pellets corresponding to 10 μg of proteins, using a 2-steps extraction protocol (Folch method) with methanol and chloroform in different proportions [16]. Briefly, ammonium bicarbonate resuspended pellets were spiked in with 16 internal standards (IS mix) and subjected to a first extraction by adding 1 mL of a mixture of chloroform and methanol 91:9 *v*/*v*. After centrifugation at 9300× *g* for 10 min, the organic phase was recovered. On the aqueous phase, a second extraction was performed with 1 mL of chloroform and methanol 66:33 *v*/*v*, after 45 min of incubation, the organic phase was recovered, combined with the previous one and dried out in a Speedvac to be then finally resuspended in 50 μL of buffer A. Internal standards were spiked in the samples at the concentrations listed in Table 1. All the operations were performed on ice.

As an alternative, lipids were extracted using a single-step extraction protocol with methanol and chloroform. Briefly, water resuspended pellets were made up to 170 μL with water and spiked in with 1 μL of SPLASH^®^ LIPIDOMIX^®^ Mass Spec Standard. The lipid extraction consisted in adding 700 μL of methanol followed by a sonication of 1 min at 4 °C, and subsequently an addition of 350 μL of chloroform. Samples were mixed on the orbital shaker for 15 min at 4 °C. After that, a further addition of 350 μL of water/chloroform (1:1 *v*/*v*) were added to each suspension and centrifuged 10,000× *g* for 10 min at 4 °C. The organic phase was recovered and dried out in a Speedvac to be then finally resuspended in 50 μL of ethanol and buffer A 10:90 *v*/*v*.

### 3.5. Liquid Chromatography

Lipids were separated by micro and nano LC chromatography. For microflow analysis, lipids extracts were resuspended in 50 μL of methanol and 4 μL were injected on a liquid chromatography system LC Ekspert nanoLC400 (Eksigent; Singapore) set in micro configuration coupled with a Triple TOF 6600 (AB Sciex; Singapore) mass spectrometer. Chromatography was performed using an analytical column HALO C18 90A 0.5 × 50 mm, 2.7 um particle size (Eksigent, Singapore) at room temperature. The gradient was starting from 5% of mobile phase B (IPA: H_2_O 90:10; 5 mM NH_4_COOCH_3_; 0.1% HCOOH) and 95% mobile phase A (CH_3_CN:CH_3_OH:H_2_O 45:45:10; 5 mM NH_4_COOCH_3_; 0.1% HCOOH) and linearly increased to 100% B over 35 min at a flow rate of 10 μL/min. For nanoflow analysis, lipids extracts were resuspended in 50 μL of methanol or in 50 μL of a buffer composed by 95% of mobile phase A (ACN:H_2_O 40:60; 5 mM NH_4_COOCH_3_; 0.1% HCOOH) and 5% of mobile phase B (IPA:H_2_O 90:10; 5 mM NH_4_COOCH_3_; 0.1% HCOOH), 1 μL was diluted 1:5 and injected on the same liquid chromatography system nLC Ekspert nanoLC400 set in nano configuration coupled with the Triple TOF 6600. Chromatography was performed using an in-house packed nanocolumn Kinetex EVO C18, 1.7 μm, 100 A (Phenomenex, Torrance, CA, USA), 0.75 × 100 mm at room temperature. The gradient started at 5% of mobile phase B and was linearly increased to 100% B in 5 min, maintained for 45 min, then returned to the initial ratio in 2 min and maintained for 8 min at a flow rate of 150 nL/min.

### 3.6. Mass Spectrometry Analysis

The samples were analyzed in technical duplicate, in positive mode with electrospray ionization. Data acquisition and processing were performed with Analyst TF (version 1.7.1, AB SCIEX, Foster City, CA, USA). For microflow analysis the following parameters were used: CUR 30 psi, GAS1 30 psi, GAS2 30 psi, source temperature 200 °C, capillary voltage 5500 V. Spectra were acquired by full-mass scan from 200–1800 *m/z* and information-dependent acquisition (IDA) from 100–1800 *m/z* (top 10 spectra per cycle). The de-clustering potential was fixed at 80 eV, and the collision energy was fixed at 40 eV, target ions were excluded for 20 sec after 2 occurrences. For nanoflow the following parameters were used: CUR 10 psi, GAS1 0 psi, GAS2 0 psi, source temperature 80 °C, capillary voltage 2000 V. Spectra were acquired by full-mass scan from 200 to 1700 *m/z* and information-dependent acquisition (IDA) from 50 to 1800 *m/z* (top 8 spectra per cycle). The de-clustering potential was fixed at 80 eV, and the collision energy was fixed at 40 eV, target ions were excluded for 20 s after 2 occurrences.

### 3.7. Data Processing and Analysis

Semi-targeted lipidomics analysis was performed by using LipidView (version 1.3 beta, AB SCIEX, Foster City, CA, USA). Lipid identification was based on exact mass, retention time, and MS/MS pattern. Lipid species based on precursor fragment ions were determined using a comprehensive targets list in LipidView. Lipid species identification was performed using a mass tolerance of 0.05 Da both in MS and in MS/MS, s/n of 3 and % peak intensity > 0 for positive ion mode. Lipid classes included for statistics and downstream analysis were: cholesterol ester (CE), sphingomyelin (SM), diacylglycerol (DAG), triacylglycerol (TAG), ceramide (Cer) phos-phatidylcholine (PC), phosphatidylethanolamine (PE), phosphatidylglycerol (PG), phosphatidylinositol (PI), phosphatidylserine (PS) and lysophosphatidylcholine (LPC), lysophosphatidylethanolamine (LPE), lyso-phosphatidylglycerol (LPG), lysophosphatidylinositol (LPI), lysophosphatidylserine (LPS), hexosylceramide (HexCer), dihexosylceramide (Hex2Cer), trihexosylceramide (Hex3Cer), sulphatides (SGalCer), ceramide-phosphate (CerP). Peak areas of Internal standards were obtained by using PeakView (AB SCIEX, Foster City, CA, USA). Untargeted lipidomics analysis was performed by using SMfinder [26]. The processing parameters were set as follows: resolution of 30,000 with deisotoping option for the peak picker; 120 s retention time tolerance for “Unique ID”; 10 ppm error with exclusion of halogenated formulas for MS analysis; blind library with forces association and filter hierarchy based on FDR and ppm, and minimum count of 3 for the filler function. The databases for the untargeted analysis were HMDB [36], MoNA [37], LipidBlast [38] and METLIN [39]. SMfinder database formats are also available at https://www.ifom.eu/SMfinder/library.php (accessed on 15 September 2021) [40].

### 3.8. Statistics

The number of samples and the statistical tests are specified in the legends of each figure. A *p*-value < 0.05 was considered statistically significant.

## 4. Conclusions

In the present work, we presented a sensitive nanoLC method, Opti-nQL, that shows high reproducibility and robustness for lipidomics identification and quantitation. Up to now, only few nanoLC methods have been used in lipidomics [17,41] and they all point to a better performance compared to the most widely used microLC ones. Our method covers the majority of lipid classes, using only one polarity mode, thus lowering the analysis time compared to other methods. Moreover, Opti-nQL shows higher sensitivity and enables the identification of more than 700 lipids, species starting from a lipid extract equivalent to 40 ng of cellular proteins. Here, we used the Folch extraction method as it is widely adopted [22] but we have also adopted a single-step extraction method able to improve qualitatively and quantitatevely the lipid extraction, highlighting the Opti-nQL versatility. Moreover, we showed that Opti-nQL is highly stable both in terms of lipid quantitation and retention time variability making it very attractive for routine lipidomics analysis.

A limitation of the method remains the analysis of coeluting isobaric species that might be overcome by using Opti-nQL with novel techniques based on ion mobility separation coupled to MS analysis that have the capability to resolve coeluting isobaric compounds. However, these methods are still limited considering the few updated lipids database based on cross collisional section (CCS) values, thus requiring in-house developed libraries and specialized instruments [34].

In conclusion, nanoLC lipidomics is highly versatile, might be easily applied in many laboratories, and enables a comprehensive and robust lipidomics characterization which might be further enhanced with the implementation of lipids databases. We strongly believe that this method might be beneficial for lipidomic studies, both in basic and biomedical research.

## Figures and Tables

**Table 1:** List of internal standards used for lipidomics analysis.

Class	Subclass	Species	Concentration
Glycero-Phospholipids	PG	PG 12:0/13:0	7.5 pmol
	PI	PI 12:0/13:0	54 pmol
	PE	PE 12:0/13:0	52 pmol
	PS	PS 12:0/13:0	43 pmol
	PC	PC 12:0/13:0,	40 pmol
Sphingolipids	CER	Ceramide d18:1/25:0	100 pmol
	GlcCer	GalCer d18:1/12:0	50 pmol
	LacCer	LacCer d18:1/12:0	50 pmol
	Sa	Sphinganine (d17:0)	50 pmol
	S	Sphingosine (d17:1)	50 pmol
	S1P	Sphingosine-1-P (d17:1)	100 pmol
	GalSph	Galactosyl(s) Sphingosine-d5	20 pmol
Glycerolipids	DAG	D5-DAG ISTD Mix I	20 pmol
	TAG	D5-TAG ISTD Mix I	20 pmol
Sterol Lipids	Chol	Chol-d7	800 pmol
	CE	CE (19:0)	100 pmol

## Data Availability

Data available within the article.

## Supplementary Materials

The following are available online at https://www.mdpi.com/article/10.3390/metabo11110720/s1, Figure S1: Salt crystallization evaluation. Figure S2: Total ion chromatogram (TIC) of Internal Standards (IS) mix at different concentrations. Figure S3: Internal standards linearity evaluation. Figure S4: Internal standards intra- and inter-day variability. Figure S5: Internal standards profile in MEFs and IPSC samples, Table S1: Peak areas of the lipid species identified by LipidView for lipidomics analysis of MEFs cells in micro flow LCMSMS. Table S2: Peak areas of the lipid species identified by LipidView for lipidomics analysis of MEFs cells in nano flow LCMSMS. Table S3: Peak areas of the lipid species identified by LipidView for lipidomics analysis of IPSC cells in micro flow LCMSMS. Table S4: Peak areas of the lipid species identified by LipidView for lipidomics analysis of IPSC cells in nano flow LCMSMS. Table S5: Peak areas of the lipid species identified by LipidView for lipidomics analysis of WM115 cells in nano flow LCMSMS, Table S6: lipids identified in IPSC by SMFinder. Table S7: lipids identified in WM115 by SMFinder. Table S8: lipids identified in MEF by SMFinder.
