# Supplementary material for: Opti-nQL: An Optimized, Versatile and Sensitive Nano-LC Method for MS-Based Lipidomics Analysis
Source: Metabolites. 2021 Oct 21;11(11):720. doi: 10.3390/metabo11110720 (PMC8623082; doi:10.3390/metabo11110720)
Supplement: Supplementary file 1 [file metabolites-11-00720-s001.zip › Supplementary/Cattaneo etal_supplementary_rev2 to zip.pdf]

Opti-nQL: an optimized, versatile and sensitive nano-LC method for MS-based lipidomics analysis

Angela Cattaneo[1], Giuseppe Martano[2], Umberto Restuccia[3,4], Laura Tronci[3,5] , Michele Bianchi[1], Angela Bachi[3*ǂ], Vittoria Matafora[3ǂ]

[1] Cogentech SRL Benefit Corporation, 20139 Milan, Italy.
[2] Institute of Neurosciences, CNR, 20133 Milan, Italy
[3] IFOM, The FIRC Institute of Molecular Oncology, 20139 Milan, Italy.
[4] Present address: ADIENNE Pharma & Biotech, Caponago, Italy
[5] IRCCS San Raffaele Scientific Institute, Molecular Basis of Cystic Kidney Diseases, Division of Genetics and Cell Biology, 20132 Milan, Italy.
* Correspondence: angela.bachi@ifom.eu +39 02 574303 873. IFOM - the FIRC Institute of Molecular Oncology, via Adamello 16, 20139 Milan, Italy
ǂ These authors contributed equally

Table S1. Limit of detection (LOD) and limit of quantification (LOQ) of Internal standards. LOD and LOQ was expressed as 3.3 and 10 times respectively of the ratio between the standard deviation of the response and the slope of the calibration curve. LOD and LOQ were determined by weighted linear regression.

Lipid standard	LOD (fmol)	LOQ(fmol)	R2	SLOPE
PC(12:0/13:0)	18.41	60.75	0.9872	1495410.714
Lactosylceramide (d18:1/12:0)	42.33	139.68	0.9691	553137.7551
Ceramide (d18:1/25:0)	19.67	64.92	0.9448	11076835.2
Glucosylceramide (d18:1/12:0)	4.28	14.12	0.9956	608352.551
PE(12:0/13:0)	45.80	151.13	0.9691	335421.9388
PI(12:0/13:0)	56.55	186.63	0.9642	238864.2857
PG(12:0/13:0)	3.70	12.21	0.9913	18711.22449
Cholester(19:0)	69.12	228.10	0.9878	3320092.857
Sphinganine(d17:0)	27.80	91.74	0.9658	417341.3265
Sphingosine(d17:1)	22.23	73.36	0.9605	299355.102
Sphingosine-1-P(d17:1)	95.50	315.16	0.9552	3506.6
Gal-B-Sphingosine(d17:1)	3.66	12.08	0.9936	6058377.041
DG(14:0/0:0/14:0) (d5)	10.46	34.52	0.9919	731854.5918
DG(15:0/0:0/15:0) (d5)	14.94	49.30	0.9877	479490.3061
DG(16:0/0:0/16:0) (d5)	8.03	26.50	0.9965	632066.3265
DG(17:0/0:0/17:0) (d5)	4.91	16.21	0.9959	1395901.531
DG(19:0/0:0/19:0) (d5)	14.31	47.24	0.9821	991718.8776
DG(20:0/0:0/20:0) (d5)	2.42	7.99	0.9946	1181827.041
DG(20:2/0:0/20:2) (d5)	3.00	9.88	0.9979	2711297.449
DG(20:4/0:0/20:4) (d5)	15.21	50.20	0.9924	831782.6531
DG(20:5/0:0/20:5) (d5)	14.05	46.35	0.9866	1176214.796
TG(14:0/16:1/14:0) (d5)	3.90	12.87	0.9976	7007694.898
TG(20:5/22:6/20:5) (d5)	8.58	28.31	0.9947	7822575
TG(15:0/18:1/15:0) (d5)	1.09	3.61	0.9992	6968257.143
TG(16:0/18:0/16:0) (d5)	5.76	19.02	0.9968	27252666.84
TG(17:0/17:1/17:0) (d5)	3.88	12.79	0.9981	7515920.408
TG(19:0/12:0/19:0) (d5)	5.76	19.02	0.9968	27252666.84

TG(20:2/18:3/20:2) (d5)	2.47	8.14	0.9985	7404169.388
TG(20:4/18:2/20:4) (d5)	3.57	11.77	0.9969	7426656.122
PS(12:0/13:0)	51.01	168.33	0.9451	6313225.444
Chol-d7	681.69	2249.57	0.9803	1316296.429

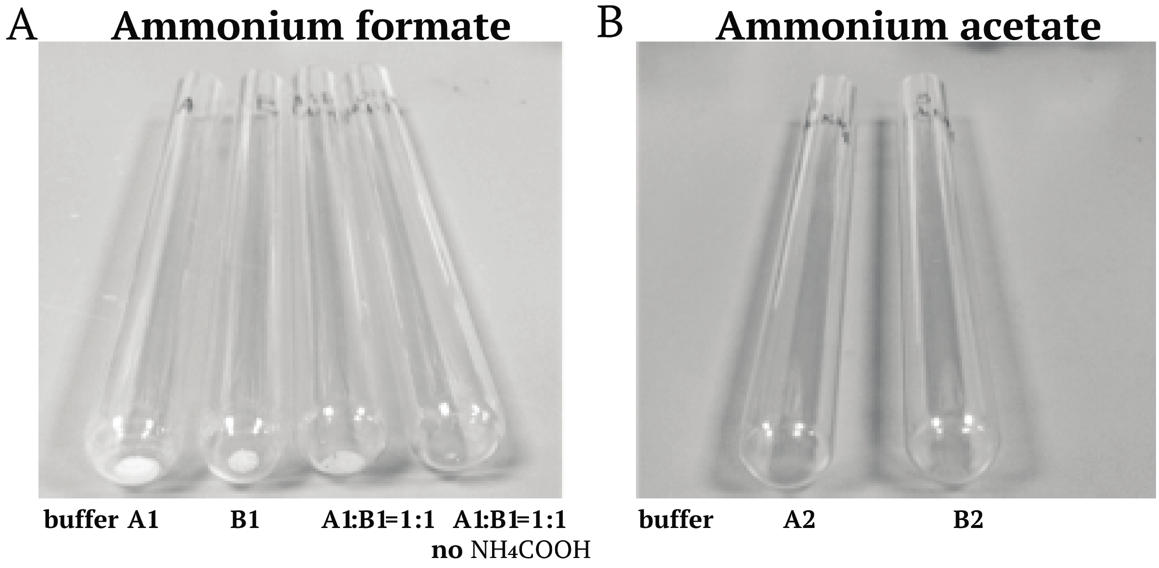

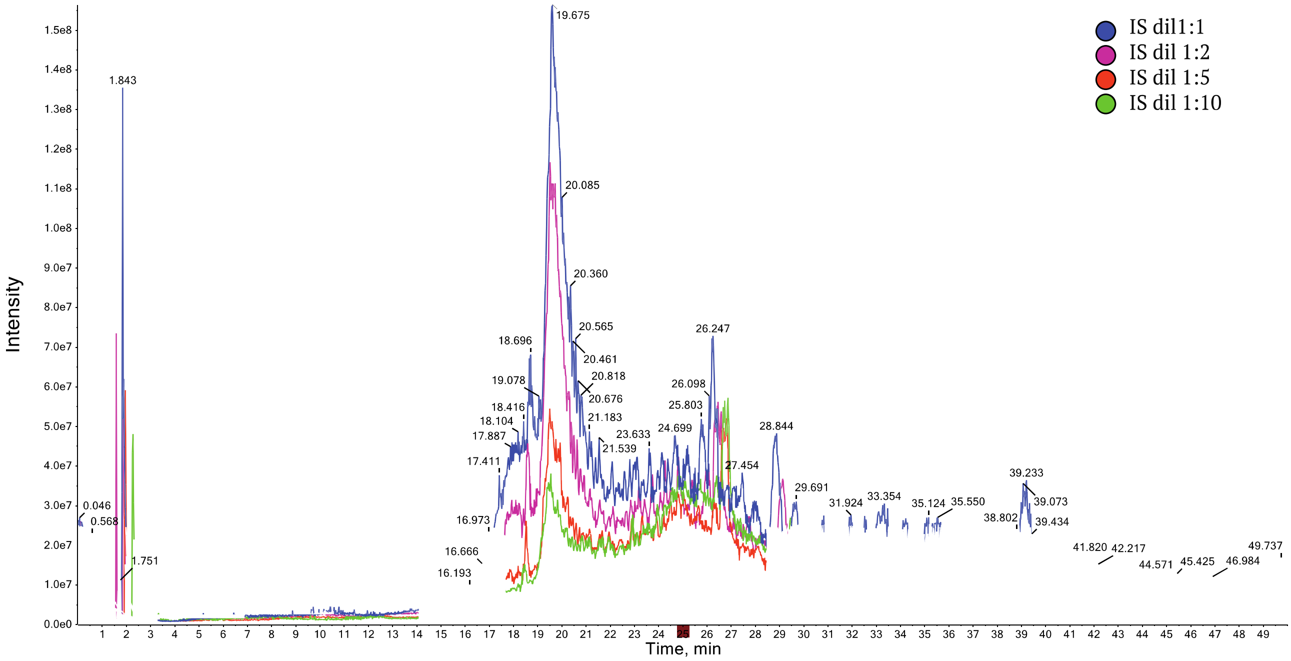

Figure S2. Total ion chromatogram (TIC) of Internal Standards (IS) mix at different concentrations. The IS mix was sequentially diluted from 1:1 to 1:10, where 1 corresponds to 1/250 of the IS concentration reported in table1, and analyzed with Peak View.

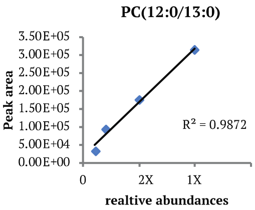

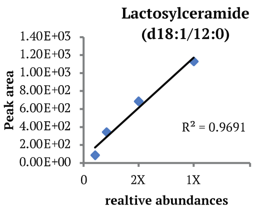

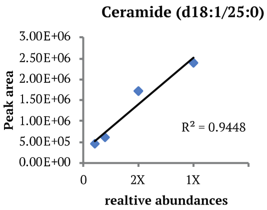

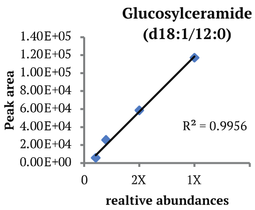

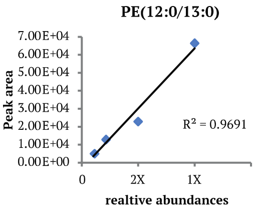

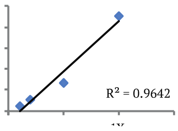

ngosine

0 2X 1X

Figure S3. Internal standards linearity evaluation. The graphs report the peak areas obtained by integrating the IS peaks with PeakView for each IS analyzed in sequential dilutions (1X-2X-5X-10X), where 1X corresponds to 1/250 of the IS concentration reported in table1. R square values indicate the linearity for each IS.

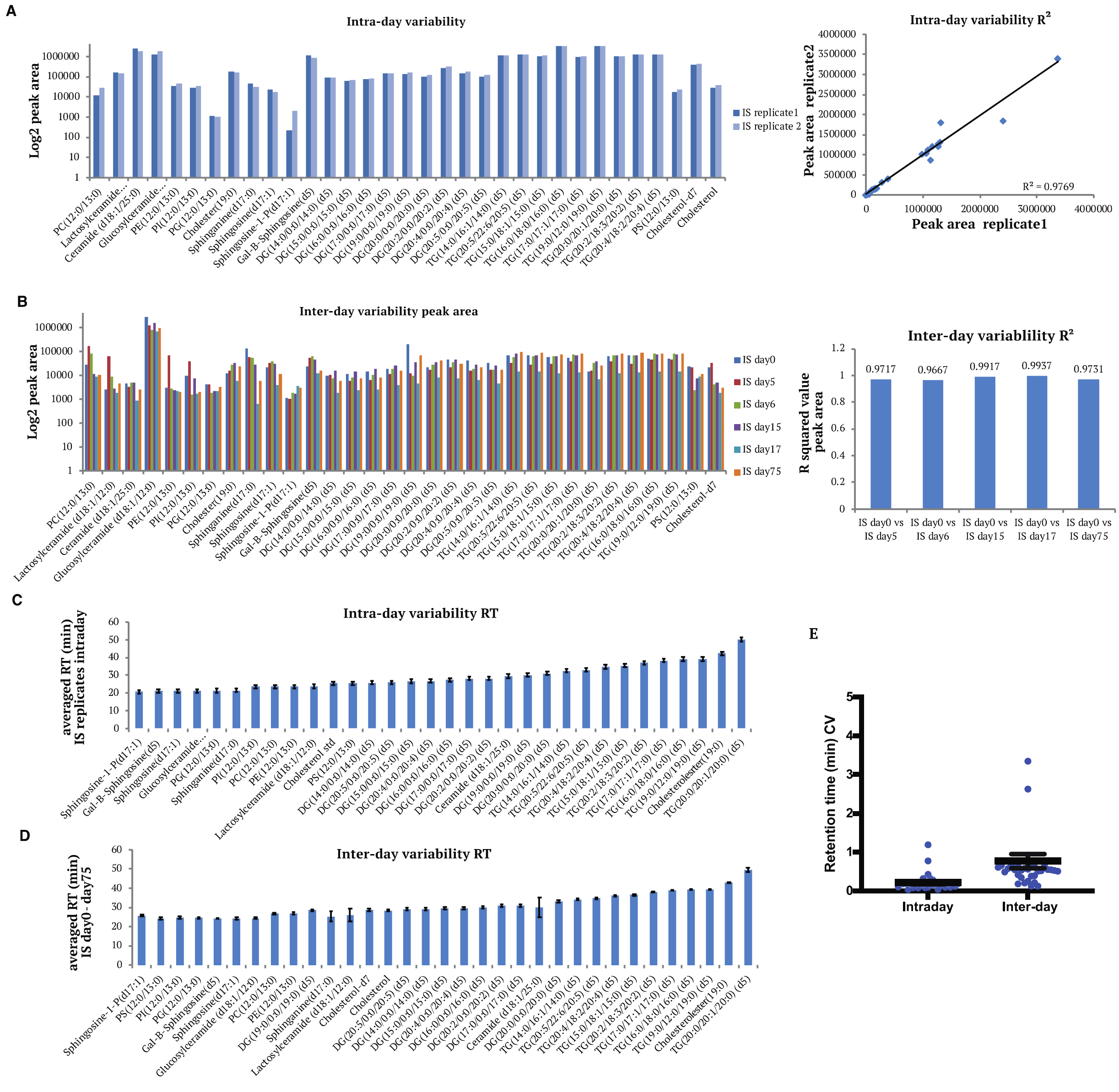

Figure S4. Internal standards intra- and inter-day variability. Panel A: intra-day variability measured as intensity of peak areas for IS replicate 1 and 2 analyzed the same day. Panel B: inter-day variability measured as intensity of peak areas for IS replicates (day 0, 5, 6, 15, 17 and 75). Panel C: intra-day variability of retention times (RT) for IS replicate 1 and replicate 2 analyzed the same day. Panel D: inter-day variability of RT for IS replicates measured at day 0 and 75. Panel E: comparison of the coefficient of variation of the retention time of each IS measured in intra-day and inter-day replicates.

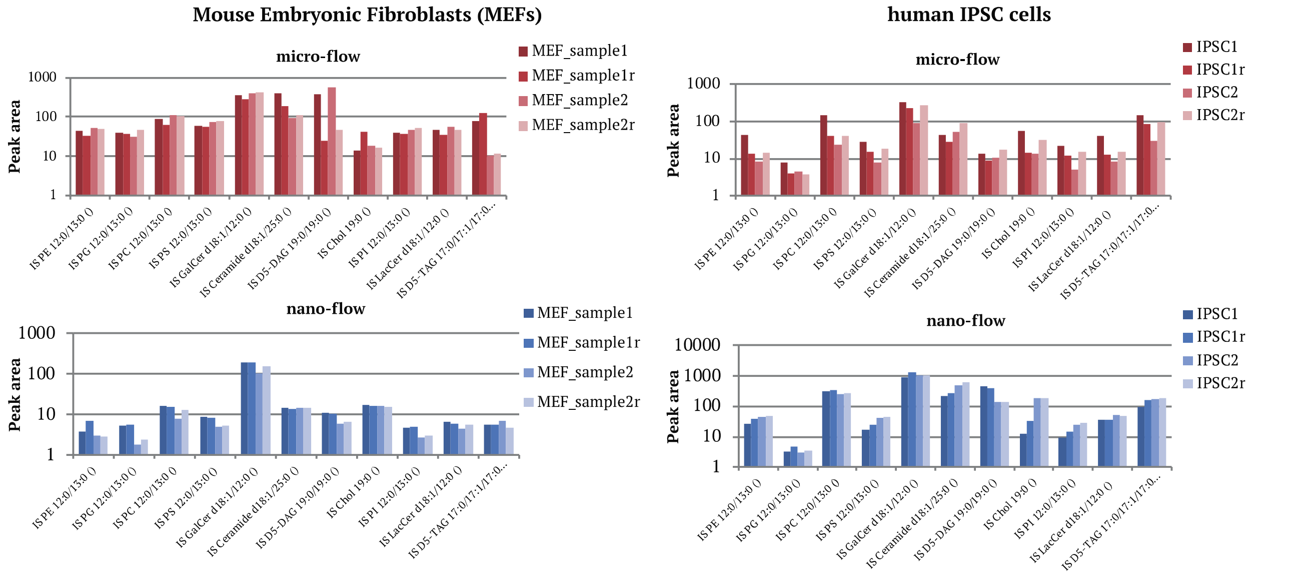

Figure S5. Internal standards profile in MEFs and IPSC samples. The Log2 of the peak area is reported for each internal standard.

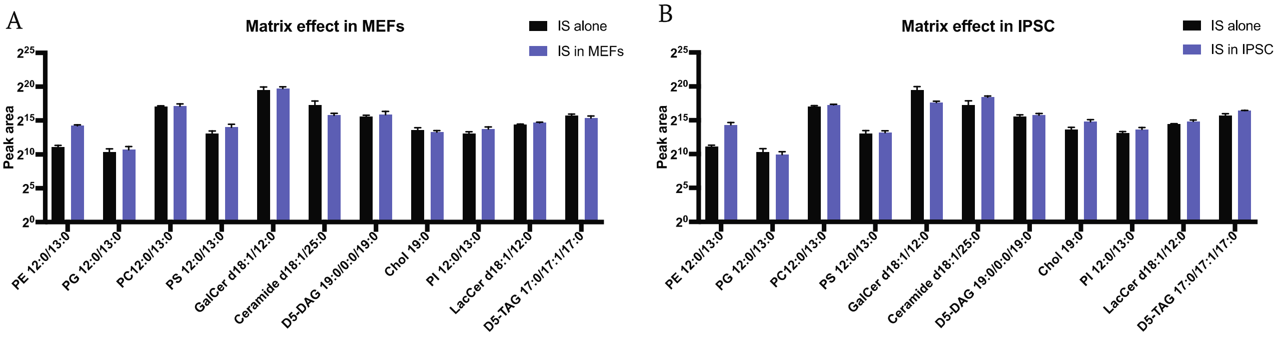

Figure S6. Matrix Effect of IS in MEFs and IPSC samples. The matrix effect was evaluated by comparing the response of the internal standards analyzed both alone and spiked into the lipid extracts of MEFs and IPSC at the same concentration. The Log2 of the peak area is reported for each internal standard.

A

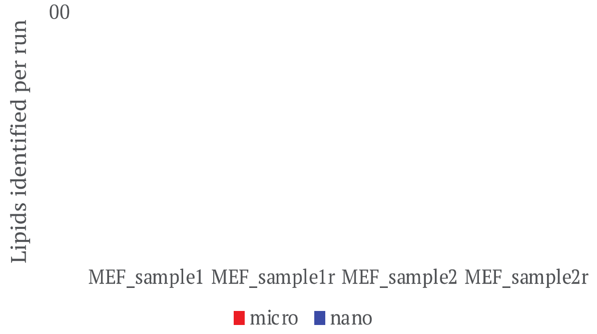

B

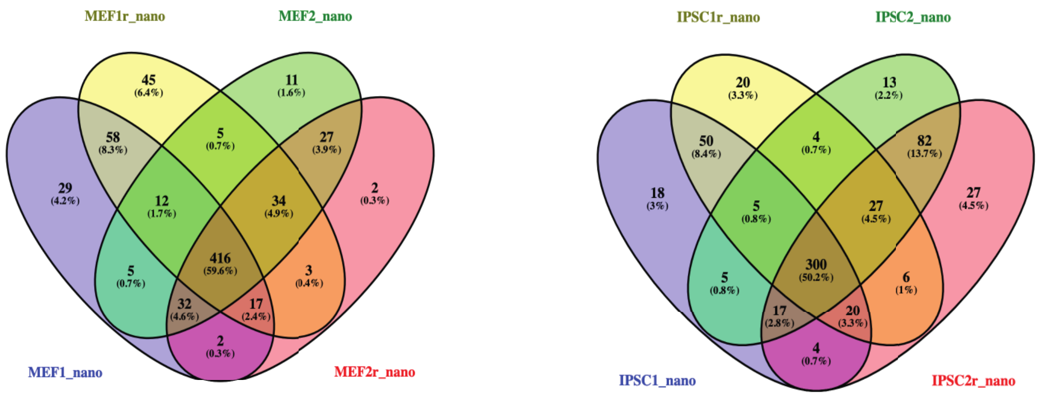

Figure S7. Lipids identified and quantified per run in MEFs and IPSC samples analyzed both in micro and nano-flow method. Panel A: Total lipids identified and quantified per run of MEFs and IPSC samples. Panel B: Venny diagram of the shared lipids identified and quantified across all the runs of MEFs and IPSC samples analyzed both in micro and nano-flow method.
